# Galvani Offset Potential and Constant-pH Simulations of Membrane Proteins

**DOI:** 10.1021/acs.jpcb.2c04593

**Published:** 2022-09-01

**Authors:** Olivier Bignucolo, Christophe Chipot, Stephan Kellenberger, Benoît Roux

**Affiliations:** † Department of Biomedical Sciences, University of Lausanne, 1015 Lausanne, Switzerland; ‡ SIB Swiss Institute of Bioinformatics, 1015 Lausanne, Switzerland; § Department of Biochemistry and Molecular Biology, The University of Chicago, Chicago, Illinois 60637, United States; ∥ Department of Chemistry, The University of Chicago, 5735 S. Ellis Ave., Chicago, Illinois 60637, United States; ⊥ Laboratoire International Associé Centre National de la Recherche Scientifique et University of Illinois at Urbana−Champaign, Unité Mixte de Recherche n◦7019, Université de Lorraine, B.P. 70239, 54506 Cedex Vandœuvre-lès-Nancy, France; # Department of Physics, University of Illinois at Urbana−Champaign, Urbana, Illinois 61820, United States

## Abstract

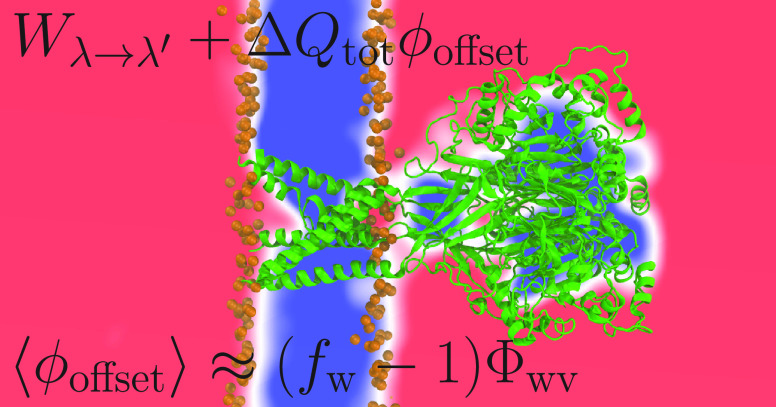

A central problem in computational biophysics is the treatment of titratable residues in molecular dynamics simulations of large biological macromolecular systems. Conventional simulation methods ascribe a fixed ionization state to titratable residues in accordance with their p*K*_a_ and the pH of the system, assuming that an effective average model will be able to capture the predominant behavior of the system. While this assumption may be justifiable in many cases, it is certainly limited, and it is important to design alternative methodologies allowing a more realistic treatment. Constant-pH simulation methods provide powerful approaches to handle titratable residues more realistically by allowing the ionization state to vary statistically during the simulation. Extending the molecular mechanical (MM) potential energy function to a family of potential functions accounting for different ionization states, constant-pH simulations are designed to sample all accessible configurations and ionization states, properly weighted according to their Boltzmann factor. Because protonation and deprotonation events correspond to a change in the total charge, difficulties arise when the long-range Coulomb interaction is treated on the basis of an idealized infinite simulation model and periodic boundary conditions with particle-mesh Ewald lattice sums. Charging free-energy calculations performed under these conditions in aqueous solution depend on the Galvani potential of the bulk water phase. This has important implications for the equilibrium and nonequilibrium constant-pH simulation methods grounded in the relative free-energy difference corresponding to the protonated and unprotonated residues. Here, the effect of the Galvani potential is clarified, and a simple practical solution is introduced to address this issue in constant-pH simulations of the acid-sensing ion channel (ASIC).

## Introduction

A central problem in classical molecular dynamics (MD) simulations of large biological systems lies in the treatment of ionizable residues. Conventional simulation methods ascribe a unique fixed ionization state to titrable residues in accordance with their p*K*_a_ and the pH of the system, assuming that this approximation can capture the predominant average behavior of the system. Constant-pH simulation schemes provide a more realistic treatment of titrable residues by allowing the ionization states to vary statistically in accordance with their proper Boltzmann weights. In practice, this approach requires extending the molecular mechanical (MM) potential energy function to account for the various discrete ionization states, accessible to the system. This is commonly done by introducing a set of coupling parameters {**λ**}, one λ for each ionizable site with λ = 0 and λ = 1 corresponding to the deprotonated and protonated states, respectively, yielding a family of potential functions, *U*(**r**, {**λ**}). Constant-pH simulation methods are designed to sample simultaneously the molecular phase space (**r**, **p**) as well as the accessible discrete ionization states of the sites according to this family of potential functions. Because the p*K*_a_ of an ionizable group is fundamentally related to the relative free energy of the protonated and deprotonated states, algorithms employed in explicit-solvent simulations must meet the challenge of assigning ionization states “on the fly” according to their proper Boltzmann statistical weight without carrying out an explicit alchemical free-energy calculations at each step. In the λ-dynamics method, Newton’s classical equations of motion are extended to allow the dynamic propagation of all the coupling parameters, λ(*t*), as a function of time *t* over a continuum of intermediate values in the interval [0, 1], such that the average population *P*(λ) can be used to reflect the proper Boltzmann weights of the deprotonated (λ = 0) and protonated (λ = 1) states of a specific site at a given pH.^1−7^ In the hybrid nonequilibrium MD/Monte Carlo (neMD/MC) constant-pH MD simulation method, the coupling parameter, λ, of a given site is progressively varied from 0 to 1 (or reversibly, from 1 to 0) as a function of time during a short trajectory, yielding a change in the ionization state that is subsequently accepted or rejected based on a generalized Metropolis–Hastings MC criterion, using the work accumulated in the course of the nonequilibrium trajectory.^8−10^ While the calculation of the nonequilibrium work in the neMD/MC algorithm relies on the continuous variation of λ, only the configurations with λ = 0 or 1 are retained, and the protonation and deprotonation of all titratable groups are sampled discretely. In contrast, the λ-dynamics algorithm treats λ as a continuous dynamical variable with a momentum within the framework of an extended Hamiltonian.

While there are several formal and technical differences between the two approaches, both share the same objective of achieving a proper Boltzmann sampling of the configurations and ionization states of the system represented by the family of potential functions, *U*(**r**, {**λ**}). Importantly, both methods are designed to rely on a statistically unbiased estimator of the relative free energies controlling the probability of the ionization states. In practice, this also means that the two methods are affected by similar issues. For instance, the free energy associated with protonation and deprotonation involves changes in the total charge in the system, known to be highly sensitive to the treatment of long-range electrostatics. The most common simulation approach consists in using an idealized infinite model built by replicating a finite box to infinity through periodic boundary conditions (PBCs),^11^ while treating the long-range interactions with the particle-mesh Ewald (PME) lattice sum.^12−16^ To maintain a neutral simulation box and a finite per-box energy under tinfoil conditions, a neutralizing charge density or “gellium” is implicitly assumed, leading to an electrostatic potential averaged over the volume of the simulation box to be strictly equal to zero at all times. Charging free-energy calculations are affected by both the PBCs and PME.^17,18^ If a simulation box contains mainly water molecules, then the mean box electrostatic potential, which corresponds to the so-called Galvani potential of the water phase, ⟨ϕ_w_⟩, is close to zero.^19,20^ More generally, the Galvani potential of the bulk water phase in a simulation box, ⟨ϕ_w_⟩, can differ significantly from this if the content of the simulation box includes large nonaqueous regions. In effect, the Galvani potential of the bulk water region is directly correlated to the volume fraction, *f*_w_, occupied by the water phase in the simulation box.^19^ Specifically, ⟨ϕ_w_⟩ ≈ (1 – *f*_w_)Φ_wv_, where Φ_wv_ denotes the water–vacuum interfacial potential.^19^ For instance, for the TIP3 water model,^21^ Φ_wv_ = −540 mV.^19,22^ Consequently, if the volume fraction of water is much smaller than 1, then the Galvani potential of the water phase will be depart from a value of zero by several millivolts. As a result, charging free-energy calculations performed with PBCs and PME in a particular simulation system are defined relative to an offset potential and must, therefore, be interpreted with care.^19,20^

These considerations about the Galvani potential of the bulk water phase have important implications for both the λ-dynamics and neMD/MC constant-pH simulation methods. In practice, both methods rely on values of ionization free energies for a set of reference model compounds in bulk solution, which are precalculated and tabulated, typically by simulating a water box with a single copy of the model compound. In such calculations, the tabulated ionization free energies are determined from simulations of a system for which the Galvani potential of the bulk water phase is essentially zero. When these same tabulated values are utilized in constant-pH simulations of a solvated protein comprising a large aqueous region, one can expect to obtain the correct free energy for the ionized side chains because the reference Galvani potential of the bulk water phase is essentially unchanged. In contrast, when the tabulated values are used in constant-pH simulations of a biomolecular system that involves large nonaqueous regions, incorrect results are obtained because the reference Galvani potential of the bulk water phase in this system is considerably smaller than zero, and as a result, the free energy of the protonated states is shifted. However, while the impact of the Galvani potential can be safely ignored in the simulation of soluble proteins when they immersed in a large bulk water phase, the situation is more delicate in the case of membrane proteins. In these systems, the volume fraction occupied by non-water regions is often considerable because of the presence of the lipid bilayer extending periodically in two dimensions.^10,23−25^ The Galvani offset potential can affect alchemical free energy computations^19,20^ as well as λ-dynamics or neMD/MC constant-pH simulations with explicit solvent and a PME treatment of electrostatics. The issue is not encountered with constant-pH simulation schemes relying entirely on implicit solvent representations^26,27^ or with hybrid-solvent schemes whereby the changes in ionization states are controlled via an implicit solvent model while conformational sampling is conducted with explicit solvent.^28,29^

**Figure 1:**
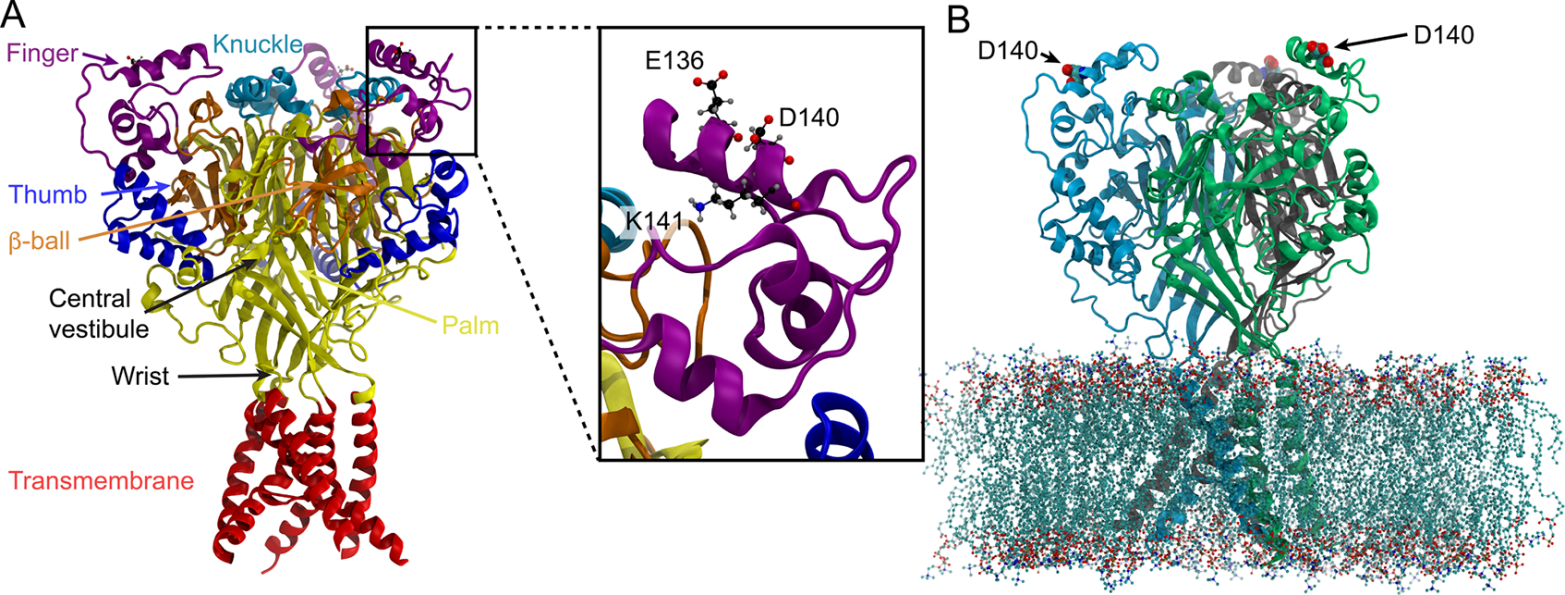
(A) Structural model of the hASIC1a showing the different parts of the protein, except for the acidic pocket, which is located between finger, thumb of a subunit, and the palm of the adjacent subunit. The model was constructed based on the crystallographic structures of the chicken structure.^39^ Inset: magnified view of the finger helix containing the Asp140 residue. The charged residues, Lys141 and Glu136, located within 3 Å of Asp140 are shown. From their orientation, one sees that they hardly interact with Asp140. (B) Molecular representation of hASIC1a inserted in a membrane containing 400 phospholipids. Each subunit is differently colored. The three Asp140 residues located in the finger region of hASIC1a are shown as spheres; two are labeled. The distance between the Asp140 of the different subunits is on the order of 59 Å.

**Figure 2:**
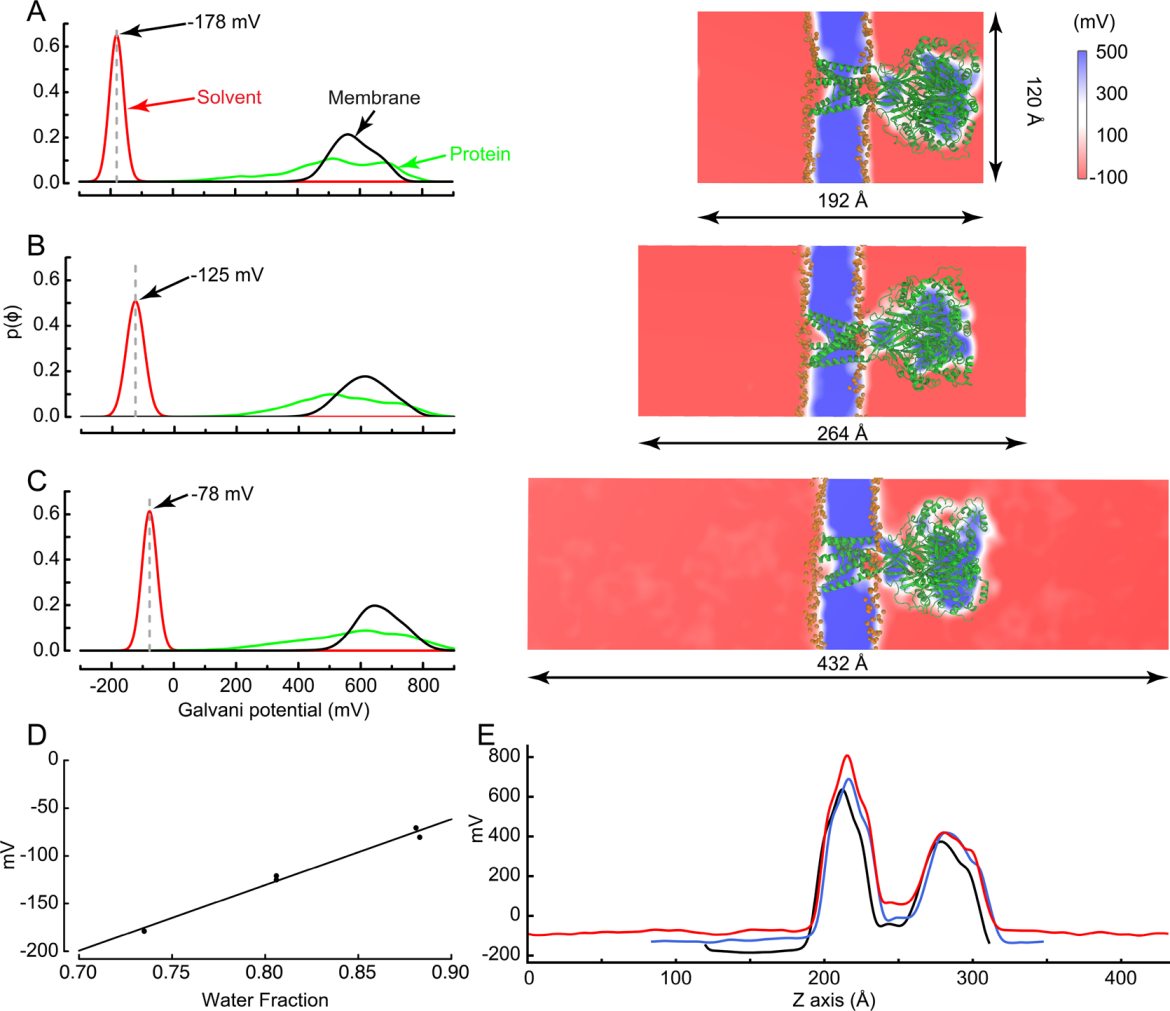
Variation of the offset Galvani potential of the bulk solvent phase as a function of the water fraction in the simulation box. (A–C, left) Galvani potential for the difference regions in the system are shown separately: bulk water (red line), protein (green line), membrane (black line), with left, probability distributions and, right, molecular representations of the three boxes. The small, medium and large simulation boxes have the same dimension in *x* and *y* (120 Å × 120 Å) but have a length of 192 Å, 264 Å, and 432 Å, along the *z* axis, respectively. The Galvani potential of the bulk water phase is −178 mV (A), −125 mV (B), and −78 mV (C) for the small, medium, and large simulation box, respectively. (D) Linear relation between the water fraction *f*_w_ and the bulk phase solvent Galvani potential ⟨ϕ_w_⟩ = (688*f*_w_ – 681) mV (fitted with *R*^2^ = 0.99, *n* = 5). (E) Potential along an axis normal to the membrane passing through the center of the small (black), medium (blue), and large simulation box (red). The potential profiles of all the simulation boxes were centered along the *Z*-axis to coincide with the structural images in parts A–C (right).

It is the goal of this work to clarify the effect of the Galvani offset potential of the bulk water phase on constant-pH simulations and implement a simple practical solution to address this issue. Our analysis will be illustrated with neMD/MC constant-pH simulations of an acid-sensing ion channel (ASIC). The ASICs are a family of H^+^-activated Na^+^ channels, which act as pH sensors in the nervous system.^30−32^ Because of their important physiological and pathological roles, demonstrated by their implication in fear behavior, learning, neurodegeneration after ischemic stroke, and pain sensation,^31,33,34^ they are potential drug targets of high interest. At physiological pH, most ASICs are in the closed state. Upon extracellular acidification, they open transiently and enter subsequently a nonconducting desensitized state. Crystal structures of chicken ASIC1a in the closed, toxin-opened, and desensitized states have been published^35,36^ as well as, more recently, cryo-EM structures of the human (h) form, thereafter called hASIC1a.^37^ It is the most abundant and most studied representative of the ASIC family.^38^ Functional ASICs are trimers, and each ASIC subunit contains a large ectodomain, two transmembrane α helices, and intracellular N- and C-termini. The structures, which resolved the transmembrane and extracellular parts, indicated that the shape of a single subunit resembles that of a hand holding a ball and that the three subunits are arranged around the central pore in a way that the hands open toward the exterior (Figure 1A). In the ectodomain, a central scaffold formed by the palm, knuckle, and β-ball as the continuation of the transmembrane segments can be distinguished from the outside-oriented parts constituted by the thumb and finger. A region called the wrist connects the transmembrane domains and the palm. An acidic pocket, so-called because of a high density of acidic residues, is located between the finger and thumb of each subunit and the palm of the adjacent subunit. The ASIC channel comprises about 1300 residues, of which about 330 are titratable. The ASIC channel is a membrane protein of considerable dimension, and a large number of lipid and water molecules are required in the simulation system to provide a realistic environment. A typical simulation system of the membrane-bound channel is shown in Figure 1B. As will be shown below, the Galvani offset potential is greatly affected by the ratio of solvent to nonsolvent volumes (Figure 2). Because of its large size, the ASIC channel provides an excellent model system to illustrate the effect of the Galvani offset potential in constant-pH simulations.

## Theory

For the sake of clarity, we first recall the theoretical formulation of our previous work in terms of a single ionizable site.^9,10^ Let **r** represent all the coordinates within the system, and *U*_p_(**r**) and *U*_u_(**r**) correspond to the potential energy function for the protonated (p) and unprotonated (u) system, respectively. The proton is converted into a dummy particle when it is noninteracting in the unprotonated system (degrees of freedom are not annihilated). To match the experimental p*K*_a_ value of a given ionizable site, it is necessary to introduce an adjustable offset constant, *C* = *k*_B_*T* ln(10) p*K*_a_ + Δ*G*, to the potential function of the protonated state, where the free-energy difference between the protonated and unprotonated states, Δ*G* = *G*_p_ – *G*_u_, is defined as
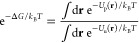
1Such a calibration is necessary because the MM potential energy function is not designed to account for true chemical bonding. To be consistent with the probability ratio of the protonated and unprotonated states from the Henderson–Hasselbalch equations, , we define the extended pH-dependent potential energy function,  in terms of the pH and the continuous coupling parameter λ, to account for the protonated (λ = 1) and unprotonated (λ = 0) states
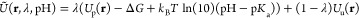
2where Δ*G* is the free energy difference of the protonated and unprotonated states for the model compound in solution. The reference free energies, Δ*G*, between the protonated and the unprotonated states of the model compounds in solution are assumed to be invariant with the sequence and the conformation of the protein. Typically, the free energies Δ*G* of the model compounds are precalculated and tabulated for efficient constant-pH simulations.

For each attempted change of ionization state in the hybrid neMD/MC constant-pH algorithm, the nonequilibrium work *W*_λ→λ′_ to protonate or deprotonate a given site is calculated through a nonequilibrium switching process through which the coupling parameter is varied from λ to λ′ and tested in the Metropolis MC criterion.^9,10^ Were this switching process done infinitely slowly, the nonequilibrium work *W*_λ→λ′_ would essentially be equivalent to the free energy of the process. Because it involves a change in the charge of a protein site, the nonequilibrium work is expected to be sensitive to the treatment of long-range electrostatic interactions in the simulation box, as would be any standard charging free energy calculation. If the protonation (λ = 0 → λ′ = 1) and the deprotonation (λ = 1 → λ′ = 0) for the protein site and for the model compound were performed with PBCs and PME in infinitely large simulation systems, then all the calculated free energies and nonequilibrium work would, by default, be constrained to the same reference potential for the bulk phase region. With simulations of finite size, the reference potential is expected to shift, depending on the content of the system.

By virtue of the tinfoil PME treatment, the **k**-space component of the Fourier transform of the potential inside the simulation box are ignored for **k** = 0,
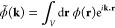
3
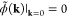
4It follows that the mean electrostatic potential averaged over the volume of the simulation box is equal to zero at all time,
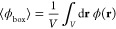
5
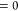
6Because the electrostatic potential averaged over the entire volume of the simulation box is constrained to zero, the mean phase potentials of the different regions in the system need to counterbalance one another (we loosely used the bracket ⟨···⟩ symbol in eq 5 to denote a spatial average over the whole volume of the simulation cell for the sake of simplicity). This introduces a constraint between the average potential of the region occupied by the bulk water phase ⟨ϕ_w_⟩ and the average potential of the remainder of the system ⟨ϕ_r_⟩. Considering a system for which the water phase has a volume fraction of *f*_w_ and the remaining non-water region corresponds to a volume fraction of *f*_r_, with *f*_w_ + *f*_r_ = 1, we have
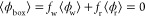
7

To make progress, let us write that
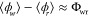
8where Φ_wr_ is some mean interfacial Galvani potential between the water phase and the non-water remainder of the system. It follows that
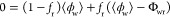
9which implies that
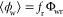
10While the preceding argument relies primarily on the unknown interfacial potential Φ_wr_, a treatment of the inhomogeneous interface of solvent with membranes and protein is nontrivial. However, the case of the water–vacuum interface can provide a good starting point to make useful approximations. A fundamental analysis shows that the water–vacuum interfacial Galvani potential, Φ_wv_, depends primarily on the trace of the quadrupole of the water molecules.^22,40,41^ For example, in the case of the TIP3 water model,^21^ the water–vacuum Galvani potential, ΔΦ_wv_, is negative in the aqueous phase and amounts to about −540 mV.^19,22^ Using Φ_wv_ as a proxy together with eq 10 to make an order of magnitude argument about the mean potential of the aqueous phase in complex biomolecular simulation systems, we obtain ⟨ϕ_w_⟩ ≈ *f*_r_Φ_wv_. This estimate shows why calculated charging free energies and nonequilibrium work calculated in finite simulation systems, where *f*_w_ ≠ 1, implicitly assume a different reference potential for the bulk water phase. Despite its obvious simplicity, this argument qualitatively captures the most important concept: the mean electrostatic potential of the water phase in a given simulation box is predominantly set by its volume fraction. The general trend displayed by the interfacial Galvani potential is remarkably robust and can actually be extended from vacuum to any non-water phases, such as the interior of a protein or a membrane.

## Method and Computations

### Atomic Model of the ASIC Transmembrane Protein

The structural model of hASIC1a was previously constructed via homology modeling^39^ based on the available crystal structures of the chicken structure in the closed state (pdb id 5WKU),^35^ with which it shares 90% sequence identity. The hASIC1a model comprises 1260 residues. Using the CHARMM-GUI server,^42^ the modeled protein was embedded in a membrane of 200 phospholipids in each leaflet, resulting in a square membrane patch spanning 120 × 120 Å^2^. In all models, the membrane normal is oriented along the *Z*-axis and the center of the bilayer is at *Z* = 0. A typical molecular representation of the channel and the membrane is shown in Figure 1.

To generate simulation systems harboring different solvent fractions, the total height of the box was set to values of 192, 264, and 432 Å. To determine whether the lipid composition affects the analysis, two different systems were used for each of the two largest boxes: a membrane containing exclusively the neutral 1-palmitoyl-2-oleoyl-*sn*-glycero-3-phosphocholine (POPC) and a membrane composed of 150 POPC and 50 negatively charged 1-palmitoyl-2-oleoyl-*sn*-glycero-3-phosphatidylglycerol (POPG) molecules. Thus, a total of five systems were simulated. These simulation systems correspond to a range of water volume fractions *f*_w_ going from 0.65 to 0.85. The 192 Å box, referred hereafter as “small”, essentially corresponds to the default setting proposed by the CHARMM-GUI membrane builder. Details of the simulation systems are given in Table 1.

**Table 1:** Molecular Systems for HASIC1A

simulations^a^		no. of atoms	no. of waters	box height (Å)
ASIC + POPC	small	262729	63121	192
ASIC + POPC	medium	359695	95383	264
ASIC + POPC/POPG	medium	359765	95383	264
ASIC + POPC	large	582206	169414	432
ASIC + POPC/POPG	large	579924	168632	432

a The composition of the membrane indicated as POPC contains 200 lipids per leaflet, and the membrane indicated as POPC + POPG is composed of 150 POPC and 50 POPG per leaflet.

#### Galvani Offset Potential in the Membrane–Protein System

While the analysis from the Theory section helps explain the main qualitative features of the electrostatic landscape of a periodic system simulated with tinfoil PME, in practice the mean potential of the bulk water phase in the simulation box ⟨ϕ_w_⟩ is calculated more accurately by using the PMEPot and VolMap plugins of VMD.^43^ The Galvani potential of the bulk water phase in the ASIC simulation systems was determined from all-atom trajectories of 35–58 ns. Such relatively short trajectories, which preclude large conformational changes of the channel while sampling the rapid dynamics of water molecules and counterions in the bulk phase, are sufficient to accurately calculate ⟨ϕ⟩ at all points **r** inside the simulation box. The simulations were generated at constant pressure and constant temperature, in the isothermal–isobaric ensemble. The following procedure was used to handle the variations in the dimensions of the simulation box and determine the electrostatics map with PMEpot. A combination of Python scripts and the VMD PMEPot and VolMap plugins was used for the analysis, considering a grid of 1 Å spacing allowed us to ascribe the Galvani potential to different regions and, thus, attribute the mean electrostatic potential value to either the solvent, protein, or membrane.^43^ To do so, we first extracted for each individual frame the dimensions and the coordinates of the center of the box and calculated the electrostatic potential of each 1 Å cubic cell in a three-dimensional grid constructed over this frame. In a second instance, with the purpose to assign to each cell of the grid one of the three species in the box (solvent, protein, or membrane), using the VolMap VMD plugin, we extracted the three-dimensional distribution of each molecular species (solvent, protein, or membrane) at 1 Å resolution. By combining these two processes, we attributed to each individual cell of the grid one of the three molecular species. The results are summarized in Figure 2 and Table 2.

**Table 2:** Galvani Offset Potential and Water Fraction

simulations		⟨ϕ_w_⟩ (mV)	*f*_water_
ASIC + POPC	small	–178	0.74
ASIC + POPC	medium	–125	0.81
ASIC + POPC/POPG	medium	–121	0.81
ASIC + POPC	large	–78	0.88
ASIC + POPC/POPG	large	–71	0.88

### Molecular Dynamics Simulations

All simulations were performed by using ver. 2.13 of the NAMD program.^44^ The CHARMM36 force field was used for all components.^45−47^ The TIP3P water model^21^ was used, and the bulk phase was modeled as a 150 mM NaCl ionic solution. Electrostatic interactions were treated with PME,^12^ and a real-space cutoff of 12 Å with a smooth switching region was used for the electrostatic and Lennard-Jones interactions. All bonds involving hydrogen atoms were kept rigid with the SHAKE algorithm,^48^ allowing a time step of 2 fs. The temperature and pressure were maintained at 310 K and 1 atm by using respectively Langevin dynamics and the Langevin piston.^49^ Default ionization states were ascribed to all titratable residues. Configurations from the trajectories were saved at a 40 ps interval.

Constant-pH simulations with the neMD/MC algorithm were performed to examine the titration of Asp140—a solvent-exposed residue located in the finger region of the hASIC1a channel (Figure 1B). Assuming that there is no long-range allosteric coupling between the three subunits, no correlation would be expected between the Asp140, as they are separated by a large distance of about 59 Å. The ionization state of the remaining residues was unchanged. The constant-pH simulations comprised 400 cycles of neMD/MC, with nonequilibrium switches of 20 ps (10000 steps) followed by 1 ps (500 steps) of equilibrium MD. The length of the equilibrium MD simulations, only 1 ps here, was deliberately kept short to focus our attention on the nonequilibrium switches. Separate constant-pH simulations were performed at six different values of the pH for the systems in pure POPC membranes to capture the titration curve around the expected p*K*_a_ of Asp140. Simulations were performed at a pH of 1, 2, 3, 4, and 5 in the presence of a Galvani potential offset correction. Simulations were performed at a pH of 3, 4, 5, 6, 7, and 8 in the absence of any correction.

The result from the different constant-pH simulations were combined by using the binless WHAM procedure.^10,50^ The MD data from the small, medium, and large simulations were processed separately. Briefly, we consider *N*_s_ independent simulations at pH_*i*_ and with Galvani potential ϕ_*i*_. There are *n*_*i*_ snapshots in the simulation *i*. The total number of protons in the system at snapshot *t* from simulation *i* is equal to *n*_*i*,*t*_ (with *t* ≤ *n*_*i*_). From this, the perturbed potential energy *U*_*j*_(**R**_*i*,*t*_) with pH_*j*_ and Galvani potential ϕ_*j*_ can be written as
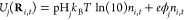
11From these definitions, the free energies {*f*_*k*_} can be computed self-consistently from
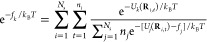
12and the average of any quantity of interest *S* for potential energy *U*_*k*_ can be computed from
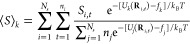
13For example, one can calculate the average protonation state of any residue as a function of pH and Galvani potential by using a given set of constant-pH simulations. In practice, to generate the full titration curve, it is convenient to first create a list from the MD data for the *N*_s_ simulations, with their number of snapshots , pH’s , and Galvani potentials , and then append to this a list of desired values of pH’s and Galvani potentials with zero snapshots . The entire set can be processed simultaneously via eq 12 to get the free energies constants {*f*_*k*_}, and the average protonation states ⟨*S*⟩_*k*_ for the pH’s {pH_*k*_} and Galvani potentials {ϕ_*k*_} can be calculated via eq 13. For independent sites, the WHAM treatment yields a titration curve akin to the Henderson−Hasselbalch equation.

### Continuum Electrostatic Calculations

To offer a comparison, the p*K*_a_ of Asp140 was also examined by using a continuum electrostatic approximation in which the solvent is represented implicitly. The continuum electrostatic calculations were performed by using the Poisson–Boltzmann equation (PBEQ) finite-difference solver^51^ included as a module in the CHARMM program.^52^ A simulations of 10 ns with deprotonated Asp140 was performed, and a frame was extracted every 2 ns for PBEQ calculations. Default ionization states were ascribed to all other titratable residues. The conformation of the side chain in the protein was kept unchanged for the reference calculation of the side chain in solution. A dielectric constant of 80 and a salt concentration of 0.15 M were ascribed to the continuum solvent. The protein–solvent dielectric boundary was constructed by using a set of optimized atomic Born radii.^53^ The thickness of the membrane (around 40 Å) and the level of insertion of the protein were extracted from the frames. A dielectric constant of 8 was ascribed to the phospholipid headgroups. The total electrostatic potential was calculated at each point of a discrete grid by solving the finite-difference PB equation. The calculation was performed in two steps, first by using a grid spacing of 1.5 Å followed by a focusing around the main region with a grid spacing of 0.5 Å. The resulting p*K*_a_ of Asp140 was 3.98 ± 0.3 from this procedure.

## Results and Discussion

### Galvani Potential for the hASIC1a Channel Simulation System

The average potential of the bulk water phase in the simulation box, ⟨ϕ_w_⟩, was estimated directly by using the PMEPot and VolMap plugins of VMD.^43^ The histogram of the value of the potential at all grid points is shown in Figure 2A.

The position of the highest peak, which corresponds to the largest region in the simulation system, was used to determine the mean Galvani potential of the bulk water phase region. Identifying the relevant offset potential from the highest peak is preferable than trying to perform a spatial average over the solvent region because the regions close to the protein or membrane are not, for structural reasons, expected to accurately reflect mean Galvani potential of the bulk water phase. The results in Figure 2D show that the Galvani potential of the bulk water phase follows the empirical relation ⟨ϕ_w_⟩ = (688*f*_w_ – 681) mV. The slope differs from the 540 mV expected for a water–vacuum interface by about 27%. In simulations of solvated lipid bilayers, estimates of the water–membrane interfacial potential tend to be around 650–700 mV, which is slightly larger than the water−vacuum interfacial potential Φ_wv_.^54^ The Galvani potential in the interior of the hydrocarbon region of the bilayer appears, in effect, to be slightly more positive than vacuum relative to bulk water. Nonetheless, as shown by the results in Table 2, the Galvani potential of the bulk phase ⟨ϕ_w_⟩ is primarily determined by the volume fraction of water *f*_w_ and is not very sensitive to the membrane composition. The changes attributable to the different membrane compositions, pure DOPC to versus POPC/POPG, are on the order of 4 and 7 mV for the medium and large systems.

### Constant-pH Simulations of the hASIC1a Channel

The calibration of constant-pH simulation methods with explicit solvent must first consider the charging free energy associated with the protonation and deprotonation processes for model compounds in bulk solution.^1−10^ In these bulk systems, the volume fraction occupied by water molecules in the simulation box is almost equal to 1, and the mean Galvani potential of the bulk phase, ⟨ϕ_w_⟩, is essentially equal to 0 mV. This implicitly establishes the reference state potential for all model compounds and subsequent constant-pH simulations. However, this reference is not respected with a simulation box comprising large macromolecular structures such as the membrane-bound hASIC1a channel (Figure 1B). In this case, the total volume fraction occupied by the nonaqueous regions is much larger than 0. For this reason, the magnitude of the Galvani potential of the bulk water phase can depart considerably from 0 mV.

The solution that we propose to resolve this issue is to introduce an offset correction, ϕ_offset_ = −⟨ϕ_w_⟩, to bring back the Galvani potential of the bulk phase to a value of 0 mV. By virtue of the Galvani offset potential correction, the proper reference potential used in the simulations of the model compound is recovered. With the correction, the work calculated from a nonequilibrium switch becomes *W*_λ→λ′_ + Δ*Q*_tot_ϕ_offset_, where Δ*Q*_tot_ is the total charge increment resulting from the change in protonation state. For a single ionizable site, Δ*Q*_tot_ = ±*e* for a protonation or deprotonation, respectively. More generally, if *n* ionizable sites are modified by the switching process, then the total charge increment is Δ*Q*_tot_ = δ*ne*, where δ*n* is the net change in the number of active protons resulting from the switching process.

**Figure 3:**
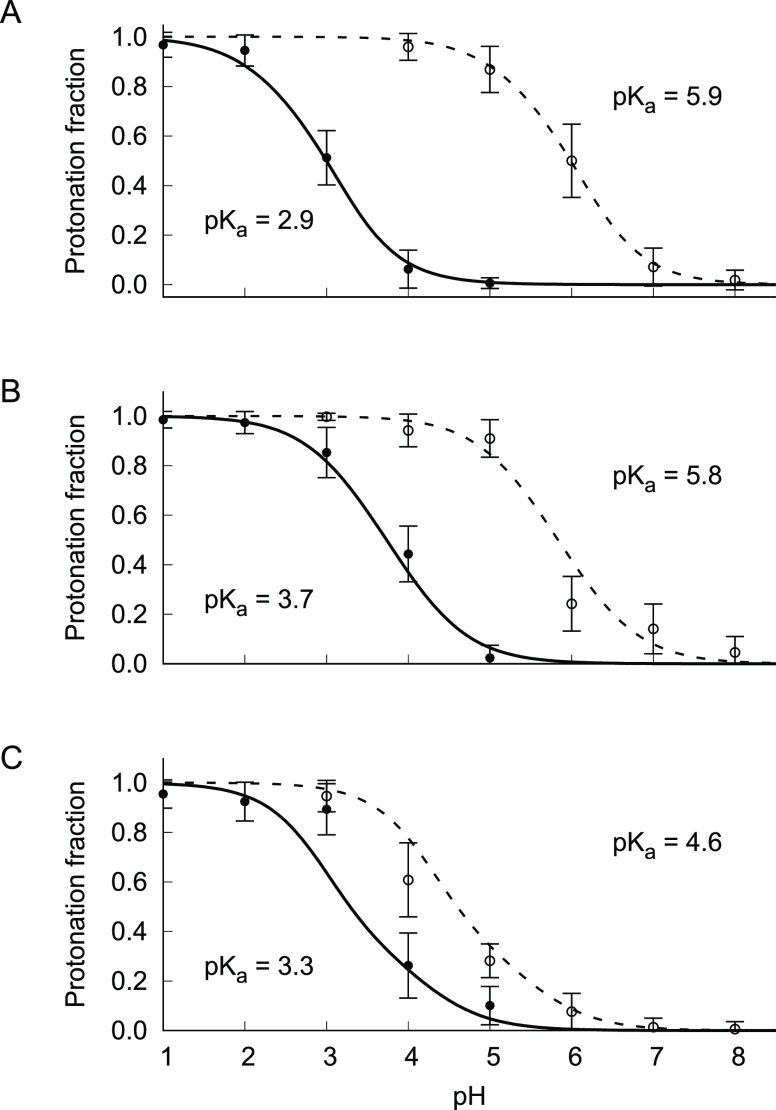
Effect of the box size and the Galvani potential correction on titration curves. For each panel, the titration of Asp140 in the hASIC1a channel is shown without correction of the potential (dashed line) or with offset correction (solid line). Shown are the protonated fractions at various pH values (mean and standard deviation, *n* = 3). The lines corresponding to the Henderson–Hasselbalch equation were obtained directly from the WHAM treatment.^10^ The calculated p*K*_a_’s and uncertainties errors are given in the insets. The different system sizes are small 192 Å box (A), medium 264 Å box (B), and large 432 Å box (C). All the systems correspond to pure POPC membranes.

To validate and illustrate the influence of the Galvani potential offset correction, we considered the titration of Asp140—a solvent-exposed residue located in the finger region of the hASIC1a channel (Figure 1B). There are three Asp140 in the hASIC1a channel, one in each of the three subunits of the trimeric protein. First, we performed constant-pH simulations with the neMD/MC algorithm in the absence of the Galvani potential offset correction. The constant-pH neMD/MC algorithm is calibrated such that the p*K*_a_ of an Asp side-chain in aqueous solution is equal to 4.0 pH units.^10^ The titration curves for Asp140 in Figure 3 show that the p*K*_a_ extracted from the titration curves vary significantly with the size of the simulation system, from 4.6 to 5.9 pH units. As the size of the simulation system decreases, the bulk water phase potential shifts toward a negative value, artificially increasing the probability to accept protonation attempts and decrease the probability of deprotonation attempts in the neMD/MC algorithm. As a result, the ionizable residues tends to be protonated with a higher probability, corresponding to an artificial upshift in the apparent p*K*_a_. While the artifact is clearly worse with the smallest simulation box, it remains an issue even with the largest simulation box. In the case of the smallest simulation box, which comprises 263000 atoms, the p*K*_a_ is shifted to 5.9 pH units (Figure 3A, dashed line). However, the value of p*K*_a_ is still upshifted to 4.6 pH unit—even for the largest simulation box comprising 582000 atoms (Figure 3C, left side). Following the relation Δp*K*_a_ = *e*Δϕ/*k*_B_*T* ln(10), a shift of 1 unit corresponds to potential difference of 59 mV. To have a deviation smaller than 0.5 p*K*_a_ unit, the Galvani potential of the bulk phase needs to be less than 30 mV. On the basis of the empirical relation ⟨ϕ_w_⟩ = (688*f*_w_ – 681) mV extracted from Figure 2D, this requires a volume fraction of at least 0.95 for the bulk water phase. The volume of the non-water region in the ASICa system is 718848 Å^3^, including the protein and the lipids. To have a water volume fraction *f*_w_ = 0.95, a volume of 13658111 Å^3^ for the water region is necessary. This value corresponds to a total of 456181 water molecules, which is considerable. This dramatically shows that attempting to resolve the issue simply by increasing the size of a membrane–protein simulation system is computationally prohibitive.

Correcting the shift in the Galvani potential of the bulk water phase with an offset correction is a practical solution to resolves this issue. As shown by the titration curves for Asp140 in Figure 3, correcting the nonequilibrium work as *W*_λ→λ′_ + Δ*Q*_tot_ϕ_offset_ consistently restores the proper free energy balance within the finite simulation systems. Upon correction of the nonequilibrium work, the three boxes now yield similar p*K*_a_ values, ranging from 2.9 to 3.7 pH units, which is smaller than the value of 4.0 in aqueous solution. The Galvani potential offset of the bulk water phase is effectively corrected. The p*K*_a_ shifts of 3.0, 2.1, and 1.3 between the corrected and uncorrected constant-pH simulations (Figure 3) are close to the predicted shifts of 2.9, 2.1, and 1.3 according to the bulk water phase Galvani potential offset of 178, 125, and 78 mV determined for the three different simulation systems (Figure 2 and Table 2). The remaining differences are most likely due to a lack of configurational sampling for these large membrane–protein systems. The difference with the p*K*_a_ in the smallest system may indicate the presence of finite-size effects. The present treatment is based on the assumption that the overall Galvani potential is fairly constant over the entire bulk water phase. Nonetheless, it is possible that dielectric and ionic screening effects are not completely effective when the volume of the bulk aqueous phase is not sufficiently large. As a comparison, the p*K*_a_ of Asp140 estimated from Poisson–Boltzmann continuum electrostatic calculations is 3.98 ± 0.3. This value is likely to be an underestimate because the conformations were taken from a trajectory generated with deprotonated Asp140, though multiple factors affect p*K*_a_ continuum electrostatic calculations.^55^ Alternative algorithms designed for the determination of the p*K*_a_ of membrane proteins could help provide additional estimates for comparison.^56^

**Figure 4:**
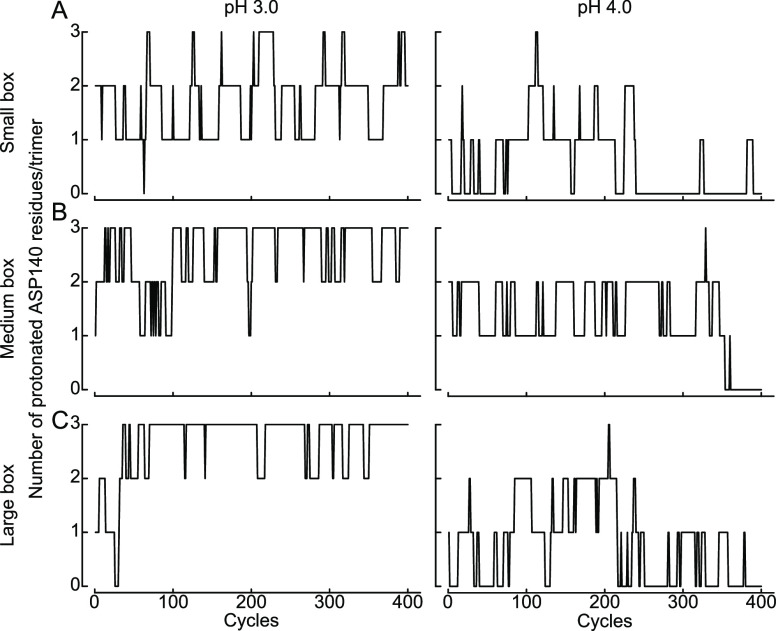
Protonation state exploration of the corrected neMD/MC algorithm. Shown are the number of protonated Asp140 residues, one per subunit of the hASIC1a trimer, as a function of neMD/MC performed cycles and at the two indicated pH values. The different system size are 192 Å box (A), 264 Å box (B), and 432 Å box (C). All the systems correspond to pure POPC membranes.

The constant-pH algorithm is functioning efficiently. According to the time series of the protonation state as a function of the neMD/MC cycles shown in Figure 4, the system appears to explore the ionization space of Asp140 during the constant-pH simulations. At a pH values around 3–4 close to the p*K*_a_ of Asp140, we observe about 50–60 switches per 400 neMD/MC cycles on average, indicating an acceptance rate on the order of 12–15%. With switches of 20 ps followed by an equilibrium step for 1 ps, this implies an accepted transition every 140 ps, which is similar to the acceptance rate of 1 transition per 50 ps observed for one isolated Asp in bulk water.^57^

In closing, it is worth discussing alternative solutions to the issue arising from the Galvani potential of the bulk water phase. Rather than determining the offset from a PMEpot analysis such as shown in Figure 2, one possibility would be to calibrate the Galvani offset potential through preliminary constant-pH simulations performed on a simple model compound like the aspartic dipeptide immersed far away in the bulk phase region of the system of interest. Assuming that the p*K*_a_ of the model compound should be unchanged, use the titration curve to calibrate the potential for subsequent constant-pH simulations.

Another strategy would be to couple all protonation/deprotonation processes with a charge-canceling counterprocess occurring in the bulk solution.^6,9,58,59^ For example, one could counterbalance the deprotonation of a residue by creating a positive charge in solution via the transformation of a water molecule into a cation or an anion into a water molecule.^6,9^ Correspondingly, one could counterbalance the protonation of a residue by creating a negative charge in solution through the transformation of a water molecule into an anion or by annihilating a positive charge in solution through the transformation of a cation into a water molecule. With a 1:1 electrolyte, such as KCl, the process requires mapping a TIP3 water molecule comprising three atoms to a monatomic ion through a standard alchemical transformation. Charge neutrality could be maintained via the simultaneous creation or annihilation of a pair of opposite charges. However, this may be less advantageous because it requires the creation of opposite charges during the alchemical switch process, yielding large energy differences that would affect the overall Metropolis acceptance probability. Coupling the protonation/deprotonation processes with counterprocesses occurring in solution is attractive, but it can also be disadvantageous because it increases the noise on the nonequilibrium work, which could decrease the acceptance probability.^9^ Chen and co-workers have also tested the idea of coupling solute titration with the charge-canceling process in constant-pH λ-dynamics simulations by coupling to ions^6^ or titratable water.^58,59^ However, the authors found that introducing additional charge-canceling coupling slows down convergence among other potential artifacts.^59^

## Conclusion

Constant-pH MD simulations with explicit solvent allowing the ionization states to vary during a trajectory represent an important addition to the set of tools that can be used to investigate complex biomolecular systems. Two well-established methods are the λ-dynamics^1−7^ and the hybrid neMD/MC.^8−10^ Despite a few formal and technical differences, both approaches aim at generating a statistically unbiased estimator of the relative free energies controlling the probability of the ionization states of the simulation system. Because changes in ionization states are associated with the total charge of the system, both approaches are sensitive to the value of the Galvani potential of the bulk water phase when used in the context of periodic boundary conditions with an Ewald lattice sum.^12−16^ While this shortcoming is of lesser concern for systems that are predominantly composed of a bulk water phase, it can become an important practical issue when the simulation box includes large nonaqueous regions.^19,20^ The problem is particularly acute as in the case of large membrane–protein systems.^10^

Here, the dependence of the Galvani potential of the bulk water phase ⟨ϕ_w_⟩ on the size of the simulation box was illustrated with the ASIC system. The value ⟨ϕ_w_⟩, varying over a range of 80–180 mV, was shown to be non-negligible even for the largest simulation system comprising almost 600000 atoms. A solution based on a Galvani potential offset correction, ϕ_offset_ = −⟨ϕ_w_⟩, was implemented to enforce a Galvani potential of the bulk phase equal to 0 mV, thus restoring the proper reference state. As an illustrative test, constant-pH simulations were generated for Asp140, a solvent-exposed residue of the protein. Simulations with the correction yield consistent results whereas simulations without the correction displayed a significant shift of the apparent p*K*_a_ of the residue.
